# Highly efficient CRISPR-Cas9-mediated editing identifies novel mechanosensitive microRNA-140 targets in primary human articular chondrocytes

**DOI:** 10.1016/j.joca.2022.01.005

**Published:** 2022-04

**Authors:** N. Chaudhry, H. Muhammad, C. Seidl, D. Downes, D.A. Young, Y. Hao, L. Zhu, T.L. Vincent

**Affiliations:** † Centre for OA Pathogenesis Versus Arthritis, Kennedy Institute of Rheumatology, University of Oxford, OX3 7FY, United Kingdom; ‡ MRC Molecular Haematology Unit, MRC Weatherall Institute of Molecular Medicine, University of Oxford, OX3 9DS, United Kingdom; § Skeletal Research Group, Biosciences Institute, Newcastle University, Central Parkway, Newcastle Upon Tyne, NE1 3BZ, United Kingdom

**Keywords:** Chondrocyte, Human, Osteoarthritis, CRISPR-Cas9, miR-140, Injury

## Abstract

**Objective:**

MicroRNA 140 (*miR-140)* is a chondrocyte-specific endogenous gene regulator implicated in osteoarthritis (OA). As mechanical injury is a primary aetiological factor in OA, we investigated *miR-140*-dependent mechanosensitive gene regulation using a novel CRISPR-Cas9 methodology in primary human chondrocytes.

**Method:**

Primary (passage 1/2) human OA chondrocytes were isolated from arthroplasty samples (six donors) and transfected with ribonuclear protein complexes or plasmids using single guide RNAs (sgRNAs) targeting *miR-140*, in combination with Cas9 endonuclease. Combinations of sgRNAs and single/double transfections were tested. Gene editing was measured by T7 endonuclease 1 (T7E1) assay. miRNA levels were confirmed by qPCR in chondrocytes and in wild type murine femoral head cartilage after acute injury. Predicted close match off-targets were examined. Mechanosensitive *miR-140* target validation was assessed in 42 injury-associated genes using TaqMan Microfluidic cards in targeted and donor-matched control chondrocytes. Identified targets were examined in RNAseq data from costal chondrocytes from *miR-140*^*−/−*^ mice.

**Results:**

High efficiency gene editing of *miR-140* (90–98%) was obtained when two sgRNAs were combined with double RNP-mediated CRISPR-Cas9 transfection. *miR-140* levels fell rapidly after femoral cartilage injury. Of the top eight *miR-140* gene targets identified (*P* < 0.01), we validated three previously identified ones (septin 2, bone morphogenetic protein 2 and fibroblast growth factor 2). Novel targets included *Agrin*, a newly recognised pro-regenerative cartilage agent, and proteins associated with retinoic acid signalling and the primary cilium.

**Conclusion:**

We describe a highly efficient CRISPR-Cas9-mediated strategy for gene editing in primary human chondrocytes and identify several novel mechanosensitive *miR-140* targets of disease relevance.

## Introduction

Osteoarthritis (OA) is generally accepted as a biologically driven disease where mechanical stresses, combined with other factors, lead to imbalance between catabolic and repair activities within the joint^1^. Multiple tissues of the joint are affected causing degradation of the articular cartilage, remodelling of subchondral bone and hypertrophy of the synovium^2^. The chondrocyte is regarded as a critical disease player; being highly mechanosensitive, able to synthesise its own degradative enzymes and having limited renewal, leading to poor tissue repair (reviewed in^3^). Multiple pathways have been associated with disease modification and many of these have been validated in murine models where the molecule of interest is deleted or suppressed pharmacologically. In recent years, exploitation of some of these pathways has been explored in clinical trials with some promising results, although we are still a way from disease modification in the clinic^4^.

MicroRNAs (*miR*s) are important regulatory molecules in all animal and plant cells. They are small endogenous RNAs, typically 20–25 nucleotides in length, that suppress specific mRNAs, by binding and targeting the mRNA for degradation or by suppressing protein translation^5^. Each *miR* is formed of a 5 prime (5p) and 3 prime (3p) strand that forms a hairpin loop. *miR*s undergo a process of maturation using two key enzymes, Drosha and Dicer, deletion of which have profound developmental phenotypes^6^^,^^7^. The mature *miR* is then loaded onto the Argonaute protein to form the active RNA-induced silencing complex^8^.

*miR*s are highly regulated in osteoarthritic cartilage and elsewhere within the joint^9^^,^^10^ and several have been investigated *in vitro* and *in vivo* with evidence of disease modification^11^, ^12^, ^13^, ^14^. One of the best studied of these is *MicroRNA 140 (miR-140)* which is specifically, and highly expressed in articular cartilage^15^^,^^16^. *miR-140* is hosted by the gene WW domain containing protein 2 (WWP2), an E3 ubiquitin ligase. Genetic deletion of either *miR-140* or *Wwp2* in mice leads to skeletal abnormalities and accelerated OA^17^. These are thought to occur by affecting catabolic activity of key pathogenic proteinases such as a disintegrin and metalloproteinase with thrombospondin motif-5 (ADAMTS5), although through distinct mechanisms^18^, ^19^, ^20^. *miR-140* influences chondrocyte proliferation by reducing Sp1, a transcription factor which controls the cell cycle regulator p15 ^21^ and is modulated by mechanically driven signals^22^. As mutations affecting *miR-140* have also been linked to skeletal abnormalities in humans^23^, these studies indicate that *miR-140* plays an important homeostatic and chondroprotective role in the developing skeleton and adult joint. *miRs* can be targeted for therapeutic gain and can themselves be used as therapeutic agents, so their biology is of particular interest^24^.

Both *miRs* and small interfering RNAs (siRNAs) bind to specific mRNAs to target them for destruction^25^. siRNAs tend to have single specific targets (designed to recognise foreign mRNAs from invading pathogens) and this has been exploited as an efficient laboratory and *in vivo* tool. *miR*s usually have multiple endogenous gene targets through which they modulate cell behaviour. A recent breakthrough molecular approach utilises clustered regularly interspaced short palindromic repeats (CRISPR), an antiviral (anti-bacteriophage) defense system of prokaryotic cells, forming part of their innate immune response^26^. CRISPR sequences bind to CRISPR-associated proteins (Cas), such as Cas9, an endonuclease, that cuts the DNA after CRISPR recognises complementary DNA sequences in association with a protospacer adjacent motif (PAM). This technology can be adapted to gene editing in eukaryotic cells by designing a CRISPR-Cas9 construct that has a single guide RNA (sgRNA) that recognizes a complementary DNA target region when it is adjacent to a PAM motif (NGG or NRG, commonly found within the mammalian genome)^27^. After endonuclease action, subsequent repair of the DNA is attempted by non-homologous end joining (NHEJ), which is error prone, and hence usually leads to gene disruption. The advantage of this approach over siRNA-mediated gene suppression, is that it targets genomic DNA and thus gene silencing is permanent and can be transferred to daughter cells.

CRISPR-Cas9 mediated gene editing of primary human chondrocytes is challenging and previous reported transfection efficiencies have varied between 16% and 70%^28^^,^^29^. To explore the effects of gene editing on chondrocyte biology it has been necessary to examine either edited chondrocyte cell lines^30^, chondrocytes derived from edited induced pluripotent stem cells (iPSCs)^31^ or using clonally expanded edited chondrocytes that are likely to have lost their chondrocytic phenotype.

In this study we optimize a method to use CRISPR-Cas 9 to drive deletion of *miR-140* in primary human articular chondrocytes with high efficiency and without affecting *WWP2* expression. We examine the regulation of *miR-140* upon *ex vivo* murine cartilage injury and explore the regulation of a number of previously described and novel targets that are relevant to chondrocyte mechanobiology and OA pathogenesis.

## Methods

**Human tissue:** Osteoarthritic human articular chondrocytes were isolated from tissue obtained from individuals undergoing unicompartmental (UKA), or total knee replacement (TKR). There were no exclusion criteria. Samples, Kellgren and Lawrence grades 3–4, were obtained from the Oxford Musculoskeletal Biobank and were collected with informed donor consent in full compliance with national and institutional ethical requirements, the UK Human Tissue Act, and the Declaration of Helsinki (HTA Licence 12,217 and Oxford REC C 09/H0606/11).

**sgRNA design:** For gene editing, the ALT-R® CRISPR-Cas9 system from Integrated DNA Technologies (IDT, Coralville, IA, USA) was applied. The Cas9 protein, trans-activating CRISPR RNA (tracrRNA) and CRISPR RNA (crRNA) were all acquired from the same company. To form a functional sgRNA duplex, 3 μl of tracrRNA (5 nmol) were mixed with 3 μl of target-specific crRNA (2 nmol) in 94 μl IDT nuclease free duplex buffer and RNA quantified by nanodrop. While the universal tracrRNA forms the backbone, the crRNA is custom designed and target-specific. The required amount was then incubated for 5 min at 95°C and slowly annealed at room temperature for 10 min. Off and on-targets were predicted using a combination of two software packages: IDT (https://eu.idtdna.com/pages) and SANGER - https://www.sanger.ac.uk/htgt/wge/find_off_targets_by_seq.

**Transfections:** For RNP transfections, sgRNA (400 ng in total) was complexed with Cas9 (1 μg) for 5 min in 50ul reduced serum medium (Opti-MEM, Gibco, NY, USA). Cas9 Plus reagent (2 μl) was added before incubation. Cas9 Plus is part of Lipofectamine CRISPRMAX (Invitrogen, CA, USA). In another tube, Lipofectamine CRISPRMAX (3.5 μl) was added to Opti-MEM (50 μl), and incubated for 5 min. The Opti-MEM media containing the sgRNA/Cas9 complex was carefully added to the Opti-MEM containing Lipofectamine CRISPRMAX and incubated for another 10 min before slowly pipetting the Cas9/sgRNA/Lipofectamine into the cell media. For double transfection, cells were left for 48 h, exchanged into 10%FBS/DMEM for 24 h, then a second round of transfection by addition of freshly prepared Opti-MEM media containing the sgRNA/Cas9 complex/Lipofectamine CRISPRMAX was performed. Cells were incubated in 10%FBS/DMEM for a further 48 h before testing.

**T7 Endonuclease 1 (T7E1) assay:** DNA was first amplified by quantitative polymerase chain reaction (qPCR) using a high-fidelity DNA Polymerase (Q5® Hot Start High-Fidelity Polymerase; New England Biolabs, Ipswich, MA, USA) and gene-specific primers (Supplementary Table 2). The T7 Endonuclease 1 (New England Biolabs, Ipswich, USA) recognises and cleaves non-perfectly matched DNA such as heteroduplexes and nicked DNA. In a first step, DNA is denatured at 95°C for 5 min, then allowed to reanneal by slowly cooling it down (95 - 85°C: −2°C/s; 85 - 25°C: −0.1°C/s), allowing heteroduplex formation between wild-type DNA and CRISPR-Cas9-mutated DNA. 2 units of T7E1, which recognizes and cleaves mismatched DNA, was added in a final step to digest heteroduplexes for 45 min at 37°C.

**Statistical analysis:** GraphPad Prism 9.2.1 was used for all statistical analyses. Data are presented as mean ± 95% confidence intervals. Normality of data was tested using Shapiro–Wilk Criteria. Data were determined to be normally distributed unless specified otherwise. For statistical significance between two groups we applied either a *t*-test, paired (for matched donor samples) or unpaired. One-way analysis of variance (ANOVA) was conducted when comparing more than two groups, followed by Tukey's test (when comparing means with every other mean) or Dunnett's test (when comparing means with control mean) for comparison between the groups. RT-qPCR expression was determined by applying log 2 formula (2^−ΔΔCT^) using housekeeping genes *RNU24* (for microRNA) or *RPLP0* (pre-printed on customised microfluidic cards). Where relevant statistical significance was defined by a *P* value of <0.05.

## Results

We first assessed the impact of two different transfection methods on isolated human osteoarthritic chondrocytes (OA hACs). Passage 1 hACs were transfected either with pX330 or using a Ribonucleoprotein (RNP) complex. The pX330 plasmid is an ∼8.5 kb plasmid with the coding sequence for Cas9 and a targeting sgRNA subcloned into it. The RNP complex consisted of the Cas9 protein and targeting sgRNA. RNP transfected and untreated control OA hACs exhibited continuous growth and increased confluency at 24 h and 48 h post transfection. However, plasmid transfected OA hACs appeared to stop proliferating and exhibited high numbers of non-adherent, typan blue positive cells indicating toxicity (Supplementary Fig. 1(A) and (B)). Immunoblots confirmed that RNP transfection of OA hACs with Cas9 had been successful post transfection (Supplementary Fig. 1(C)).

Before exploring the role of *miR-140* in hACs, we first confirmed that both arms of *miR-140 (140-*5p and *miR-140-*3p), were expressed in cells taken from OA cartilage samples from several different donors (each donor represented by a different colour) (Supplementary Fig. 2). *miR-140-*5p levels were slightly higher than *miR-140-*3p (*P* = 0.0445).

**Fig. 1:**
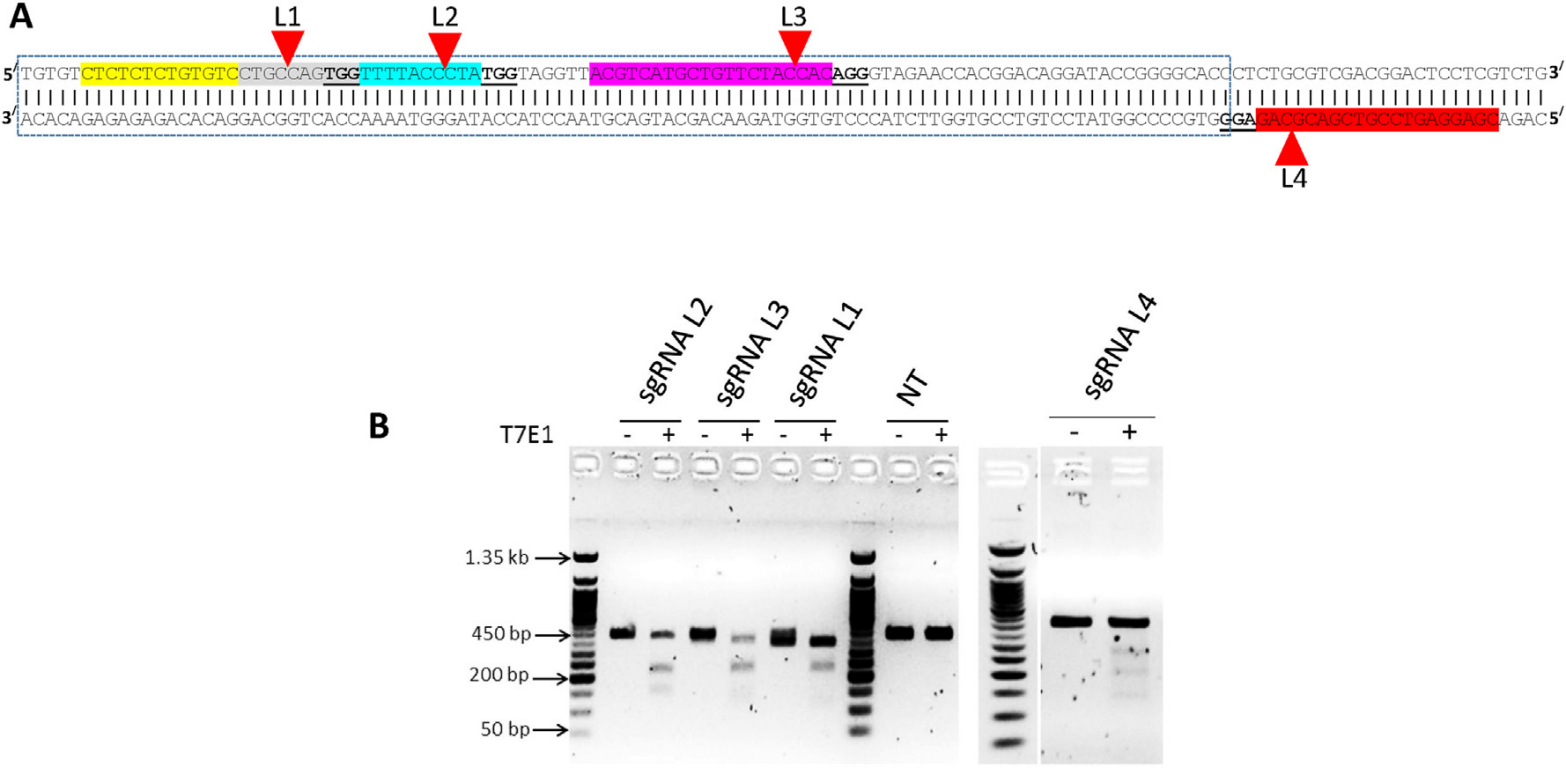
**CRISPR-Cas9 RNP transfection in primary hACs**: Sequence of genomic *miR-140* hairpin structure with positions of sgRNAs (L1, L2, L3, L4). Red arrows indicate the predicted cutting sites which cut between the 3^rd^ and 4^th^ bases from the PAM (underlined) (A). The amplified products (around 439bp) were assessed using T7E1 assay. Edited (mismatched) DNA is seen to fragment at around the predicted sizes of 180 and 260bp (B). NT, non-targeted sgRNA control. Representative agarose gel shown. *n* = 2.

**Fig. 2:**
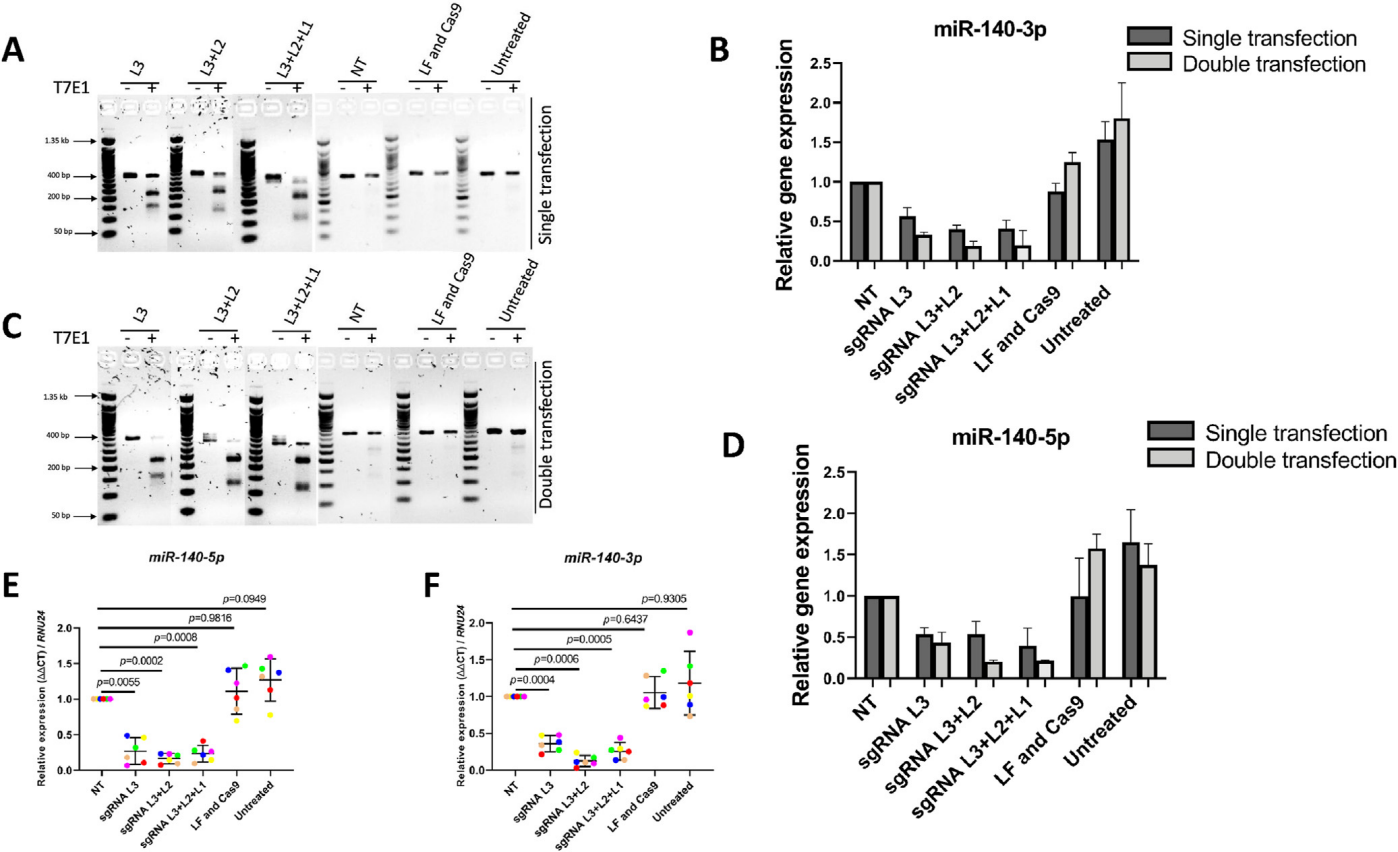
**Double transfection of CRISPR-Cas9 with RNP enhances gene editing efficiencies in hACs**. hACs underwent single or double transfection targeting individual sgRNA (L3) or combination sgRNAs (L3+L2 or L3+L2+1). Lipofectamine + Cas9 + Non-targeting sgRNA (NT), LF and Cas9 treated cells without sgRNA (LF and Cas9), or untreated cells (untreated), served as controls (A–F). T7E1 assay (A, C) and qPCR for *miR-140-*3p and *miR-140-*5p from extracted RNA *(B, D) from one donor*. Error bars are determined by technical (well) replicates (*n* = 3). Double transfection with single or combination sgRNAs was performed in a further six independent donor cells (each donor colour-coded). qPCR was performed on extracted RNA for *miR-140*-5p and *miR-140*-3p. Gene expression, a surrogate marker of editing efficiency, was normalized to NT sgRNA control and expressed relative to *RNU24.* N = 6 biological replicates (E, F). Statistical testing was performed on the raw delta CT values using the Bonferroni-Dunn method with corrections for multiple comparison.

To make the CRISPR-Cas9 RNP, four different single guide RNAs (sgRNAs), with predicted high on-target and low off-target scores, using IDT software (https://eu.idtdna.com/), were designed to target the hairpin structure of *miR-140,* which sits within an intron of *WWP2* [Fig. 1(A)]. Scores for each were as follows: L1 on-target 58, off-target 0; L2 on-target 62, off-target 59; L3 on-target 77, off-target 79; L4 on-target 40, off-target 93. From these results L2 and L3 were predicted to provide the best combination of on and off-targets. Each sgRNA was made up of a universal tracrRNA and a target specific crRNA. *miR-140* targeting sgRNAs were complexed with Cas9 as RNPs and transfected using Lipofectamine CRISPRMax. A 439 base pairs (bp) long PCR product was amplified using *miR-140* flanking primers (Fig. 1(B), dominant band running at ∼450bp). The T7 Endonuclease 1 (T7E1) assay was used to assess efficiency of CRISPR-Cas9 mediated targeting. The T7E1 assay recognises mismatched regions in double-stranded DNA (dsDNA) and cleaves the DNA at this site to generate smaller products (in this case approximately 180 and 260 bp). By T7E1 assay all four sgRNAs induced DNA fragmentation indicating successful DNA targeting, with sgRNA L4 appearing to be the least efficient [Fig. 2(B)]. This was in keeping with the low IDT scores for L4.

The impact of single or combination sgRNAs was next examined after single or double transfection. *miR-140* was targeted using sgRNA L3 (which had the highest IDT scores) or using the combination of either sgRNAs L2 and L3, or sgRNAs L1, L2 and L3, ensuring that the final concentration of sgRNA remained constant across groups. Three different controls were included: a NT sgRNA control (NT), Lipofectamine (LF) and Cas9 treated cells (no sgRNA) (LF and Cas9), as well as completely untreated cells (untreated). For each donor, each experimental condition was performed either in duplicate or triplicate. DNA cleavage was assessed by the T7E1 assay, after amplifying the 439 bp product described in Fig. 1. Gene editing was confirmed for each set of targeting sgRNAs [Fig. 2(A)] with the suggestion of improved efficiency after double transfection [Fig. 2(C)]. The impact of sgRNA on *miR-140* gene expression (a surrogate read out of editing efficiency) was examined in the same samples by qPCR. 50–60% reduction in gene expression of *miR-140-3p* [Fig. 2(B)] and *mir-140-5p* [Fig. 2(D)] was apparent after single transfection, and this was enhanced when double transfection was performed. A further six donor cells were then tested with the same combination of sgRNAs after double transfection. Reduction in the expression of both 5p and 3p arms of *miR-140* was greatest (>80%) when using the combination of sgRNAs L2+L3 (Fig. 2(E) and (F) and Supplementary Fig. 3). Cell viability, by light microscopy, appeared stable after both double and single transfections, and RNU24 raw CT values were not significantly different between transfected and non-transfected cells in either single or double transfection groups (data not shown).

Sanger Sequencing was used to confirm the deletion of bp following double transfection with sgRNAs L2+L3. Sequencing results of 15 bacterial clones, that resulted from subcloning of the PCR product, revealed that 13 out of 15 clones showed a deletion of 29 bp between the cleavage sites of sgRNA L2 and sgRNA L3 (Supplementary Fig. 4, clone 1). One clone exhibited a deletion of 31 bp, one base pair upstream of the cutting site of sgRNA L2 (highlighted in yellow) and downstream of sgRNA L3 (highlighted in blue) (clone 2). A third clone showed a deletion of 1 bp upstream of the cleavage site of sgRNA L2 and 6 bp downstream of the cleavage site of sgRNA L3 (clone 3). MiSeq analysis was used to confirm the precision of gene editing following double transfection with sgRNAs L2+L3. MiSeq analysis confirmed a deletion for over 90 % of reads (Supplementary Fig. 4(B)). Deletions of bp, in smaller numbers, were also detected upstream of amplicon position 191 and downstream of amplicon position 220. A very small number of insertions was detected at these positions. A significant majority (>97% of reads) did not exhibit any NHEJ induced bp insertion, the majority of reads (>90 %) exhibited a deletion of 29 bp or larger, with a peak at −29 bp (55 %) (Supplementary Fig. 4(C) and (D)). The absolute number of modified reads amounted to 77,094 (>99.9 %), compared with only 31 (<0.01 %) unmodified reads, suggesting very high gene editing efficiency after RNP double transfection in OA hACs (Supplementary Fig. 4(E)). To check for off-target effects of gene editing we used two different off-target algorithm software packages (see Materials and Methods). No sgRNA L3 and L2 targets with just one sequence mismatch were predicted. Two potential targets were predicted when one considered two sequence mismatches, and three when one considered three sequence mismatches. Specific primers were designed for each predicted off-target site and these were amplified in three different donors which had been double RNP transfected with sgRNA L3+L2. No evidence of DNA cleavage was seen for any of these sequences by T7E1 assay (Supplementary Fig. 5(A)). *WWP2,* the host of *miR-140* was not down-regulated following sgRNA L2+L3 relative to its normalised NT sgRNA control (supplementary Fig. 5(B)).

**Fig. 3:**
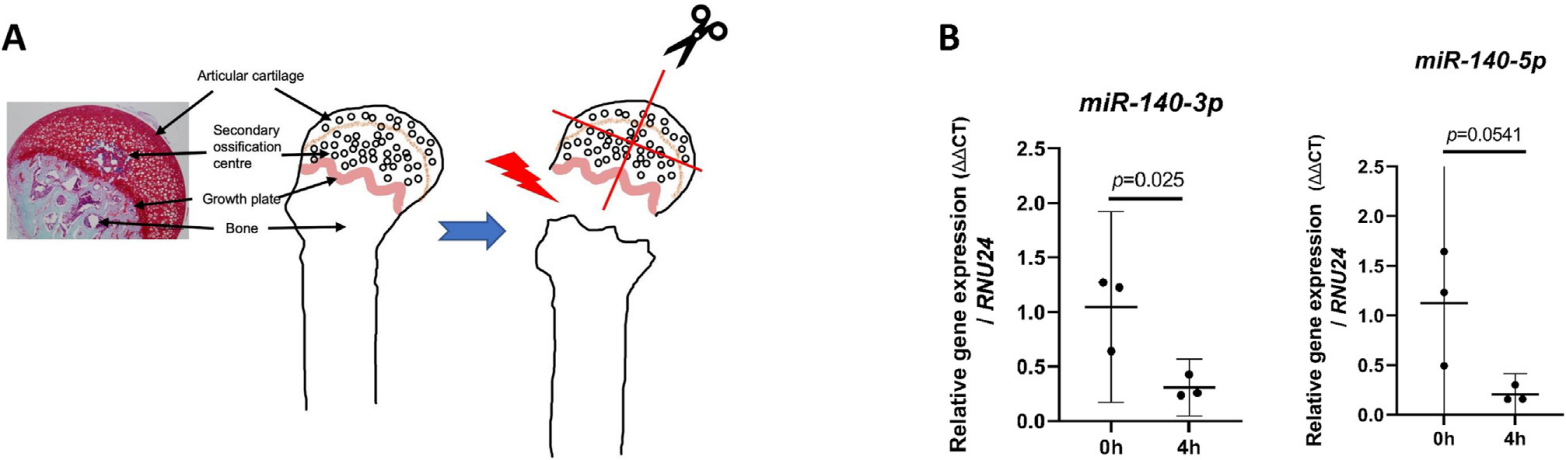
**Cartilage injury suppresses *miR-140*-3p and *miR-140*-5p expression**. (A) Safranin O/Fast Green-stained section of 6 week old murine femoral head with schematic showing the femoral head before (intact) and after cartilage injury (avulsed and cut). Femoral heads were either snap frozen immediately in liquid nitrogen to provide the negative control (0h) or cut into 4 pieces and cultured in serum-free media for 4 h at 37°C to measure the biological injury response (B) RNA was extracted and *miR-140-*3p and *miR-140-*5p expression were quantified by qPCR, normalised to *RNU24* and expressed relative to 0h. Data are shown as mean ± SD. Statistical significance by student two-tailed test. *n* = 3 biological replicates.

**Fig. 4:**
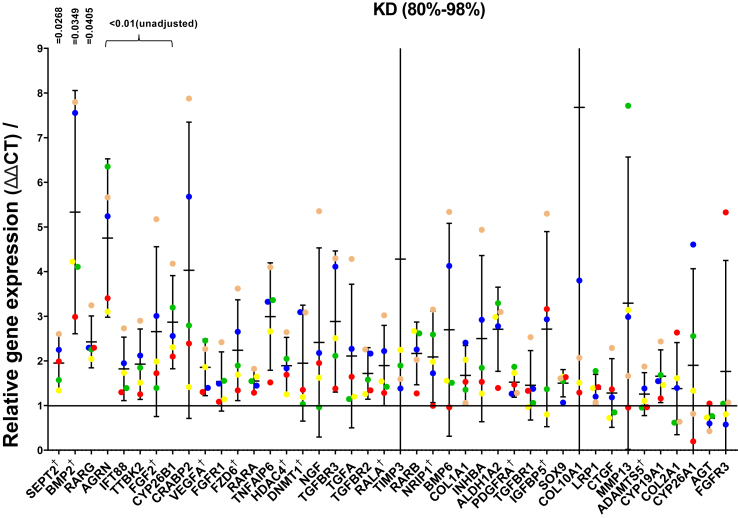
**Human chondrocyte gene editing by CRISPR-Cas9 identifies novel *miR-140* gene targets**. Gene expression of 46 genes with putative roles in osteoarthritis or previously described *miR-140* targets, were analysed by qPCR on pre-printed TaqMan Microfluidic cards. All genes were normalised to *RPLP0* and expressed relative to their respective non-targeting (NT) sgRNA control (for each donor). Genes are displayed according to the strength of the statistical significance from left to right. Gene names marked with “†” are previously identified *miR-140* targets. Each color represents an individual donor. Statistical significance was determined using the Bonferroni-Dunn method with corrections for multiple comparison. *n* = 5 biological replicates (donors). Three genes had *P* < 0.05 after correction. A further 5 genes whose uncorrected *P* values were <0.01 are also indicated.

To explore the biology of *miR-140* in articular chondrocytes we first established whether *miR-140* was regulated by cartilage injury, a critical aetiological factor in OA development and one that drives rapid changes in chondrocyte gene regulation^1^^,^^32^^,^^33^. Using a previously validated hip avulsion model in 6 week old wild type mice^33^^,^^34^ [Fig. 3(A)], both *miR-140-*5p and *miR-140-*3p were downregulated 4 h after injury (a time at which acute inflammatory genes are regulated optimally), albeit reaching statistical significance only for *miR-140-*3p *(P* = 0.0249 (95% CI -1.329, 0.1532)*; miR-140-*5*P* = 0.0541 (95% CI -1.863, 0.02596) [Fig. 3(B)]. As injury is a powerful regulator of chondrocyte gene regulation and a key etiological factor in OA development, the drop of a highly expressed *miR* could be directly contributing to the injury response. To test this, we examined a number of genes that were either previously identified *miR-140* targets or shown to regulate or to be strongly regulated by cartilage injury (Supplementary Table 1). These included genes involved in cartilage repair pathways, retinoic acid (RA) metabolism, chondrogenesis, cilia biology, and cartilage catabolism. Gene expression was examined in OA human articular chondrocytes (*n* = 5) after *miR-140* deletion and compared with their respective paired NT control. Data are presented in Fig. 4 and ranked according to p value. Each data point represents the ratio of gene expression in the deleted compared with NT control for each patient sample. *SEPT2* (1.95 ± 0.51 fold), bone morphogenetic protein 2 (*BMP2*) (5.33 ± 2.19 fold) and RA receptor gamma (*RARG)* (2.42 ± 0.47 fold) were upregulated upon *miR-140* deletion, displaying statistical significance after stringent correcting for multiple comparisons (Fig. 4). Interestingly, incomplete (50–60%) gene disruption of *miR-140* after single transfection, was insufficient to change any of the measured genes (Supplementary Fig. 6) indicating that high editing efficiency is required to demonstrate a biological effect. To validate these potential human chondrocyte *miR-140* targets further, we next interrogated RNA sequencing (RNA-seq) data taken from costal cartilage of 7-day old constitutive *miR-140* KO mice (available at NCBI GEO datasets with the accession number GSE144360). We considered the top eight genes from our qPCR analysis, whose unadjusted *P* values were <0.01 (Table I). Of these, *Sept2*, *Agrn*, *Ift88*, *Fgf2* and *Cyp26b1* showed strong regulation in *miR-140* KO costal chondrocytes (all up-regulated apart from *Ift88*). *BMP2, RARG* and *TTBK2* were not regulated in neonatal mouse costal chondrocytes by deletion of *miR-140*.

**Table I:** Genes upregulated in hACs (with *P* < 0.01) and murine neonatal costal chondrocytes upon deletion of *miR-140*

GENE	*miR-140* KD in osteoarthritic, human articular chondrocytes	P value	P value adjusted	Costal RNA from 7-day-old-*miR-140* KO mice	P value	P value adjusted	Presence of *miR-140* seed sequence
	Mean Fold change normalised to non-targeted control (lower, upper 95% CI)			Mean Fold change normalised to wild type (lower, upper 95% CI)			
*AGRN*	**4.752** (2.978, 6.525)	0.0019	0.0830	**1.641** (1.434, 1.848)	4.75E-06	9.15E-05	–
*BMP2*	**5.333** (2.609, 8.057)	0.0008	0.0349	**1.170** (0.748, 1.593)	0.9729	0.9880	140–5p
*CYP26B1*	**2.867** (1.824, 3.910)	0.0089	0.3750	**3.162** (1.985, 4.338)	1.71E-19	4.49E-17	–
*FGF2*	**2.657** (0.755, 4.558)	0.0038	0.1632	**1.523** (1.175, 1.871)	0.0033	0.0219	140–5p
*IFT88*	**1.821** (1.110, 2.531)	0.0023	0.1005	**0.675** (0.495, 0.856)	0.0002	0.0024	–
*RARG*	**2.427** (1.845, 3.009)	0.0009	0.0405	**0.845** (0.629, 1.062)	0.29772	0.5394	–
*SEPT2*	**1.95** (1.318, 2.582)	0.0006	0.0268	**1.836** (1.681, 1.990)	1.03E-11	7.81E-10	140–5p
*TTBK2*	**1.926** (1.138, 2.715)	0.0029	0.1244	**1.098** (1.004, 1.192)	0.7826	0.8992	–

Comparison of human chondrocytes after *miR-140* gene editing with genes regulated in costal chondrocytes from 7-day-old *miR-140* KO mice compared with wild type animals (determined by RNA-sequencing). Statistical testing of human data was by Student's *t*-test (two-tailed) (*P* value) with multiple comparison (*n* = 47) testing (*P* value adjusted). For the RNAseq data, we used deseq2 which uses a Wald *t*-test then a modified Benjamini Hochberg to reduce false positives. Presence of *miR-140* seed sequences indicated.

## Discussion

In this manuscript we describe a novel approach for efficient genomic editing of *miR-140* in primary human chondrocytes by performing transfection of RNP complexes containing Cas9 and sgRNA targeting sequences. This method was superior to that using the pX330 plasmid which exhibited evidence of cell toxicity within 48 h of transfection. Editing efficiency using RNP appeared to be optimal when combining more than one sgRNA, and when double transfection was performed. Efficiency at this level is usually only obtained by clonal expansion of selected targeted clones, which risks losing the chondrocyte phenotype^35^. MiSeq analysis demonstrated high gene editing efficiencies (>99.9 %) for the populations that were sequenced and qPCR indicated that *miR-140* was suppressed by >80% for all donor samples.

To explore the biological significance of *miR-140* in human chondrocytes we first assessed whether it was regulated upon cartilage injury, an important and clinically relevant stimulus for the tissue^22^^,^^36^^,^^37^. We observed that *miR-140* was downregulated 4 h following murine cartilage injury, a time at which many other genes are upregulated. *miR-*140 might therefore influence the response of the tissue to OA-induced mechanical injury and might explain the enhanced OA phenotype seen in *miR-140* knockout mice^20^. We next investigated the effect of *miR-140* deletion on chondrocyte genes that are known to be strongly regulated by injury, in addition to genes that had previously been identified as *miR-140* targets by other groups (see Supplementary Table 1and references therein). Several of these genes were upregulated in OA human articular chondrocytes upon *miR-140* deletion. None of the genes were regulated when chondrocytes were partially depleted of *miR-140* (50–60%) following a single round of transfection indicating that high level depletion is required to uncover biological function.

Considering those genes where the uncorrected *P* value was <0.01, *miR-140*-dependent genes included those involved in the RA pathway (*RARG, CYP26B1*), primary cilia biology (*SEPT2, IFT88,* and *TTBK2*), and anabolic factors (*BMP2*, *FGF2* and *AGRN)*. Several, but not all, of these were strongly *miR-140*-regulated in costal chondrocytes taken from neonatal *miR-140* knockout mice, perhaps reflecting differences in species or relating to differences in chondrocyte site and maturity (neonatal murine costal chondrocytes rather than adult human OA articular chondrocytes). *SEPT2* was the most robustly *miR-140*-regulated gene in both human articular and costal chondrocytes and has previously been identified as *miR-*140-dependent^38^. It encodes Septin 2, a filamentous GTPase, that directly binds to myosin II, a molecular motor driving muscle contraction. It is found concentrated along the axoneme (central cilium strand) in retinal pigmented epithelial (RPE) cells and deletion inhibits ciliogenesis^39^. The role of *SEPT2* in chondrocytes is unknown but it may be relevant that two other cilia-related genes (*IFT88 and TTBK2*) were also regulated by *miR-140* in human articular chondrocytes. The primary cilium has previously been linked to OA through its established role in modulating hedgehog signalling^40^. It affects aggrecanase activity in chondrocytes *in vitro* possibly by controlling the distribution of the scavenger receptor LRP1^41^, and also acts as a modulator of mechanical load in cartilage *in vivo*^42^.

Several genes with known pro-regenerative or anabolic roles were also identified as *miR-140* targets. *FGF2* was strongly *miR-140*-regulated in human articular and murine costal chondrocytes, as described previously^43^. FGF2 is released from cartilage upon injury and FGF2 deficient mice develop accelerated OA indicating its chondroprotective role^44^. Both *SEPT2* and *FGF2* possess a *miR-140-5p* seed sequence in their 3p-UTR, and so are predicted to be direct targets of *miR-140*. Two other anabolic molecules were identified as *miR-140*-dependent in human articular chondrocytes: *BMP2* and *AGRN. AGRN* is a heparan sulfate proteoglycan, usually associated with the neuromuscular junction^45^. It has recently been described as a powerful cartilage regenerative agent in damaged articular cartilage *in vivo*^46^. *AGRN*, unlike *BMP2*, was also strongly *miR-140*-dependent in neonatal murine costal chondrocytes.

We identified two novel RA regulated genes as *miR-140* targets in human articular chondrocytes (*RARG* and *CYP26B*1). *CYP26B1* was also strongly *miR-140*-dependent in murine costal chondrocytes. We have recently observed that cartilage injury strongly regulates RA dependent genes, including the *CYP26* enzymes, which are the key regulators of cellular RA levels^47^. Enhancing RA at the time of injury strongly suppresses ‘mechanoflammation’ indicating that RA is a biologically important anti-inflammatory molecule in cartilage^48^.

Contrary to previous reports^16^, *ADAMTS5* was not regulated in *miR-140* knockdown hACs in this study, even though 3′-UTR of *ADAMTS5* contains a putative seed sequence for *miR-140-3p*. Its regulation might have been uncovered had we looked at chondrocytes stimulated with IL1 or equivalent catabolic stimulus. As *miR-140* KO mice are characterised by short stature^20^, it raises the question whether the accelerated OA phenotype seen in these mice is partly due to a mild chondrodysplasia in addition to direct effects on proteases.

We recognise a number of limitations in this study. Firstly, the nature of both the T7E1 and MiSeq assays means that underestimation or overestimation of efficiency, respectively, may occur^49^. In the case of T7E1, the assay relies on DNA cleavage where there is CRISPR-Cas9 modified DNA attempting to reanneal with native DNA i.e., mismatched. If CRISPR-Cas9 mutated DNA anneals with an identically mutated strand, this will not be recognised as mismatched and therefore could result in an underestimate of gene editing efficiency. In the case of MiSeq, we identified a common polymorphic variant at amplicon position 267 (rs2102066), resulting in homology mismatch between the endogenous sequence and the reference sequence. This single nucleotide polymorphism (SNP) was present in each of the four donors, so this may account for some of the apparent NHEJ scores. This is probably exerting a small overall effect as ∼90 % of the deletions were at the predicted Cas9 target sites for the two sgRNAs for all donors. The qPCR readouts suggest the actual efficiency was between 90% and 98%. We were careful to design sgRNAs that had few predicted off-target effects, and we checked that we were not targeting sequences elsewhere within the genome with up to three mismatches. As the T7E1 assay is not highly sensitive, this result does not exclude there being low numbers of cells where off-targeting has occurred. The ability to detect off-targeting is likely to be affected, in addition, by chromatin accessibility which may be in a less open conformation in assay chondrocytes^50^. Finally, we selected a restricted number of genes of interest to explore in this study, which were, by nature, biased to those already described in the literature and of specific interest to the group.

To conclude, we have demonstrated that it is possible to get high levels of CRISPR-Cas9 mediated gene deletion in human primary articular chondrocytes. We have validated the functional effect of *miR-140* deletion by confirming previously identified targets and identifying new targets. We show, for the first time, that *miR-140* is down-regulated upon cartilage injury, and that several injury induced genes are also *miR-140* targets. This confirms the important role of *miR-140* in cartilage homeostasis, and in the injured joint in the development of OA. As chondroprotective pathways are also regulated by *miR-140*, it would not seem prudent to regard *miR-140* as a target in OA.

## Author contributions

(1) The conception and design of the study (TLV, NC, CS, DY), acquisition of data (NC, HM, DD, YH, LZ), analysis and interpretation of data (TLV, NC, DY, CS, DD, YH).
(2) Drafting the article or revising it critically for important intellectual content: all authors
(3) Final approval of the version to be submitted: all authors

## Conflict of interest

No conflicts of interest relevant to this work are identified for any of the authors.

## Statement of role of funding source

Academic funding sources did not influence the project direction or decision to publish.
