# Supplementary figures and images for: Highly efficient CRISPR-Cas9-mediated editing identifies novel mechanosensitive microRNA-140 targets in primary human articular chondrocytes
Source: Osteoarthritis Cartilage. 2022 Apr;30(4):596–604. doi: 10.1016/j.joca.2022.01.005 (PMC8987936; doi:10.1016/j.joca.2022.01.005)

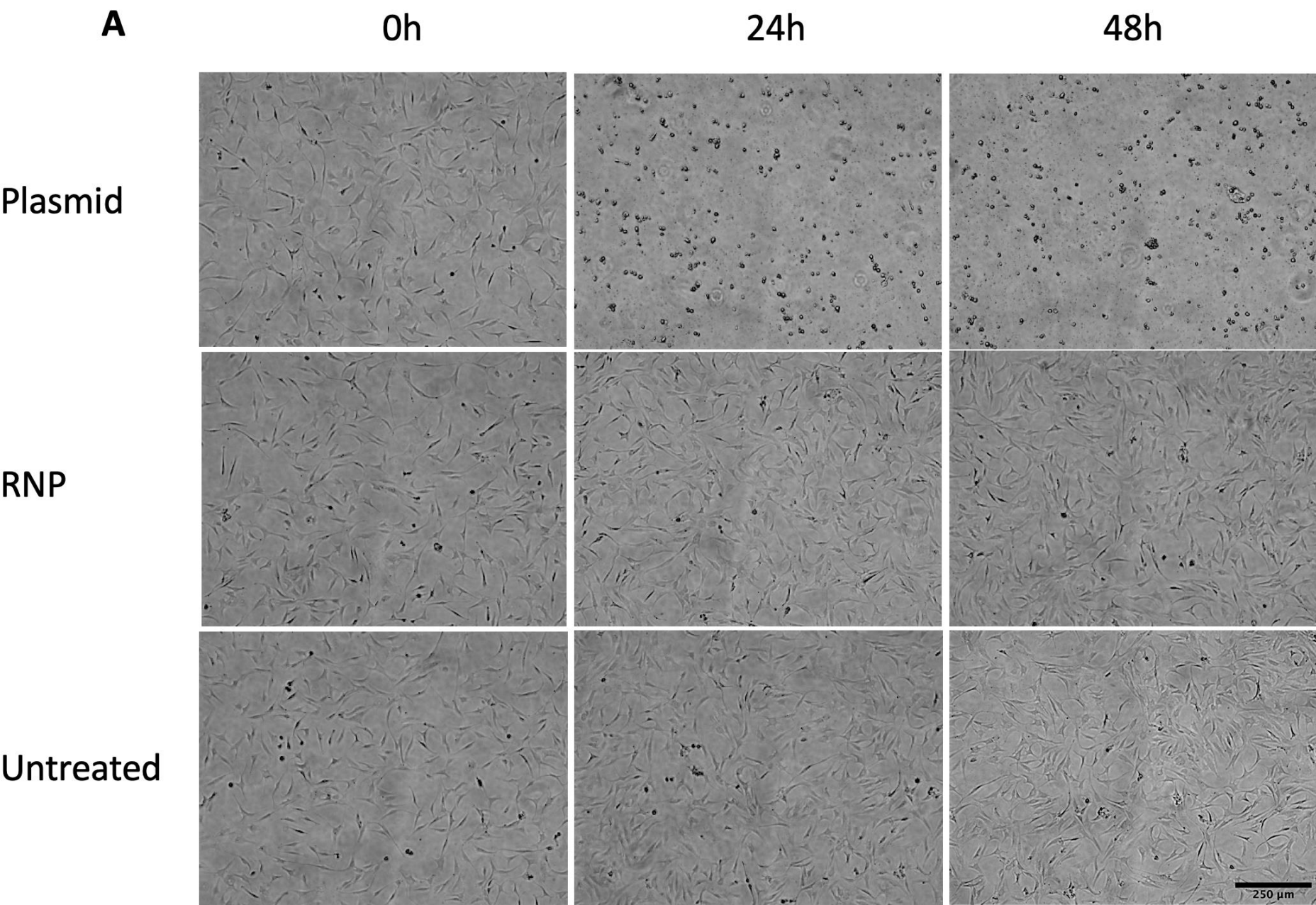

**B**

Cell death 24h post transfection

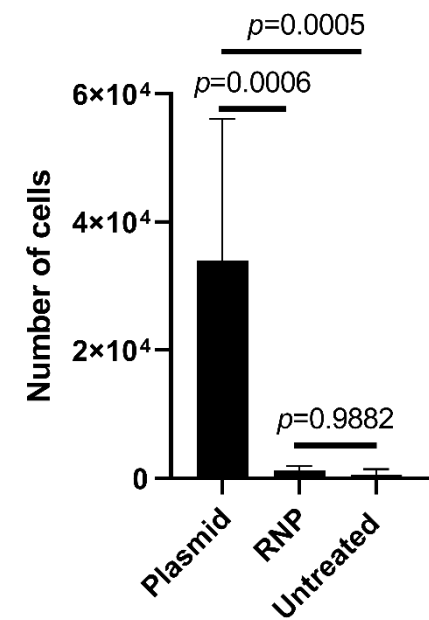

**C**

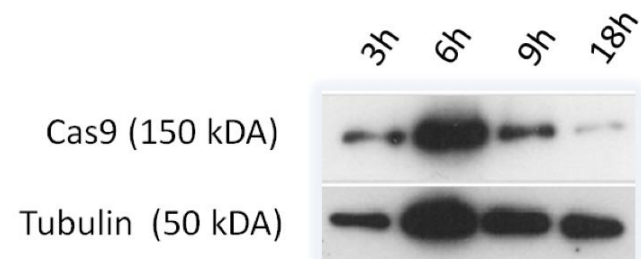

Supplementary Figure 1

Supplement: Multimedia component 1 [file mmc1.pdf]

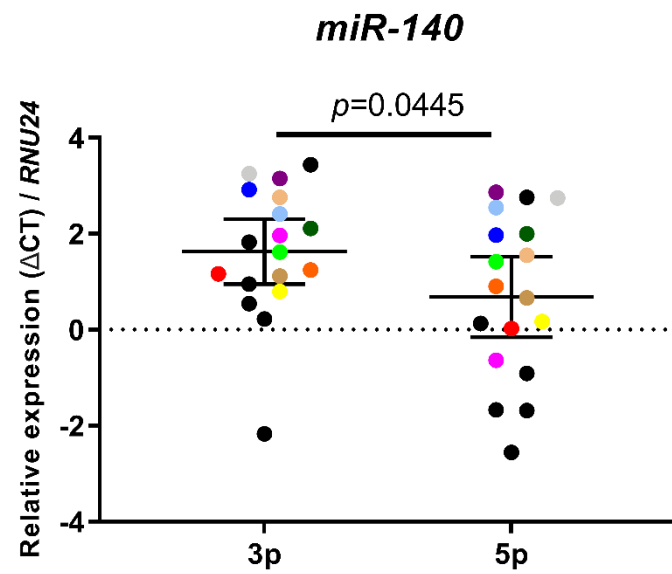

Supplementary Figure 2

Supplement: Multimedia component 2 [file mmc2.pdf]

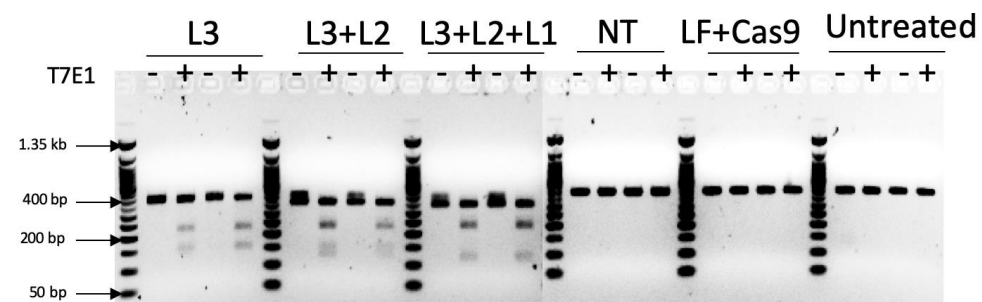

Donor 1

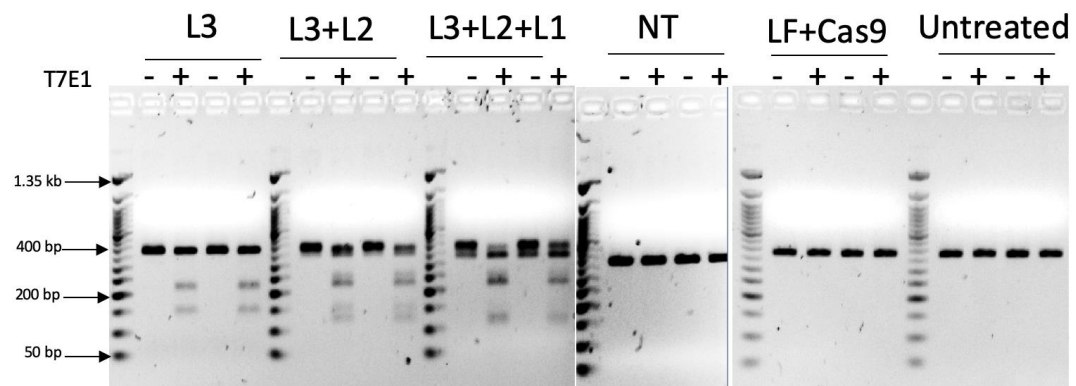

Donor 2

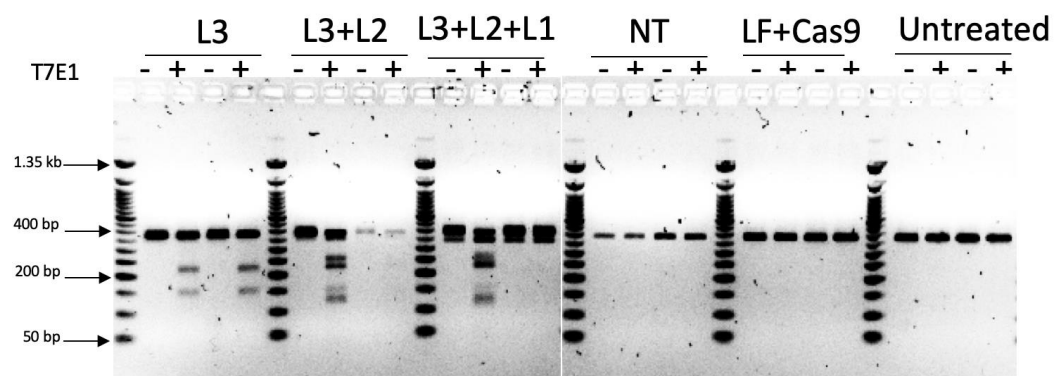

Donor 3

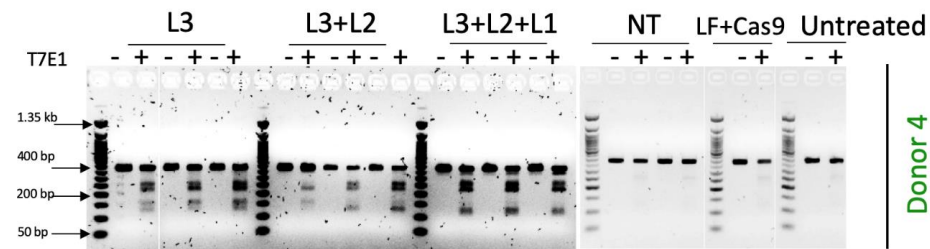

Donor 4

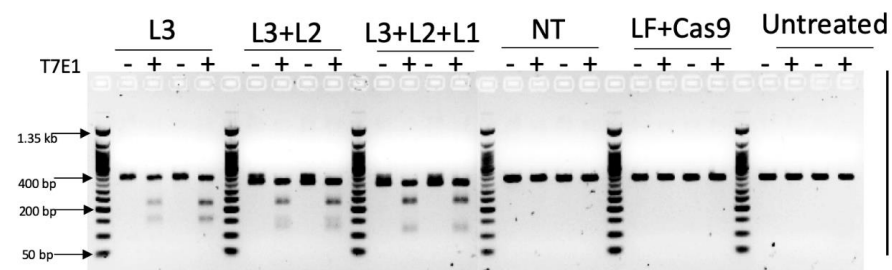

Donor 5

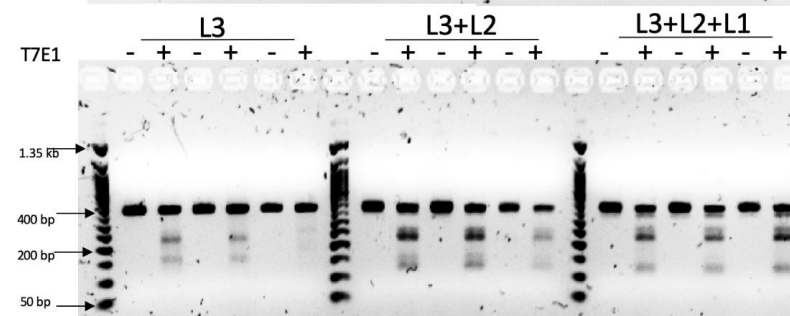

Donor 6

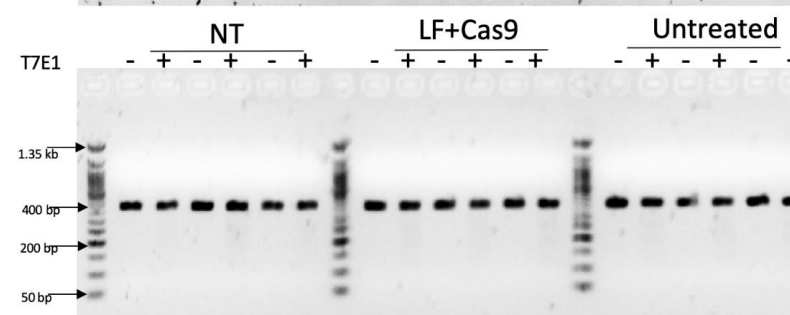

Supplementary Figure 3

Supplement: Multimedia component 3 [file mmc3.pdf]

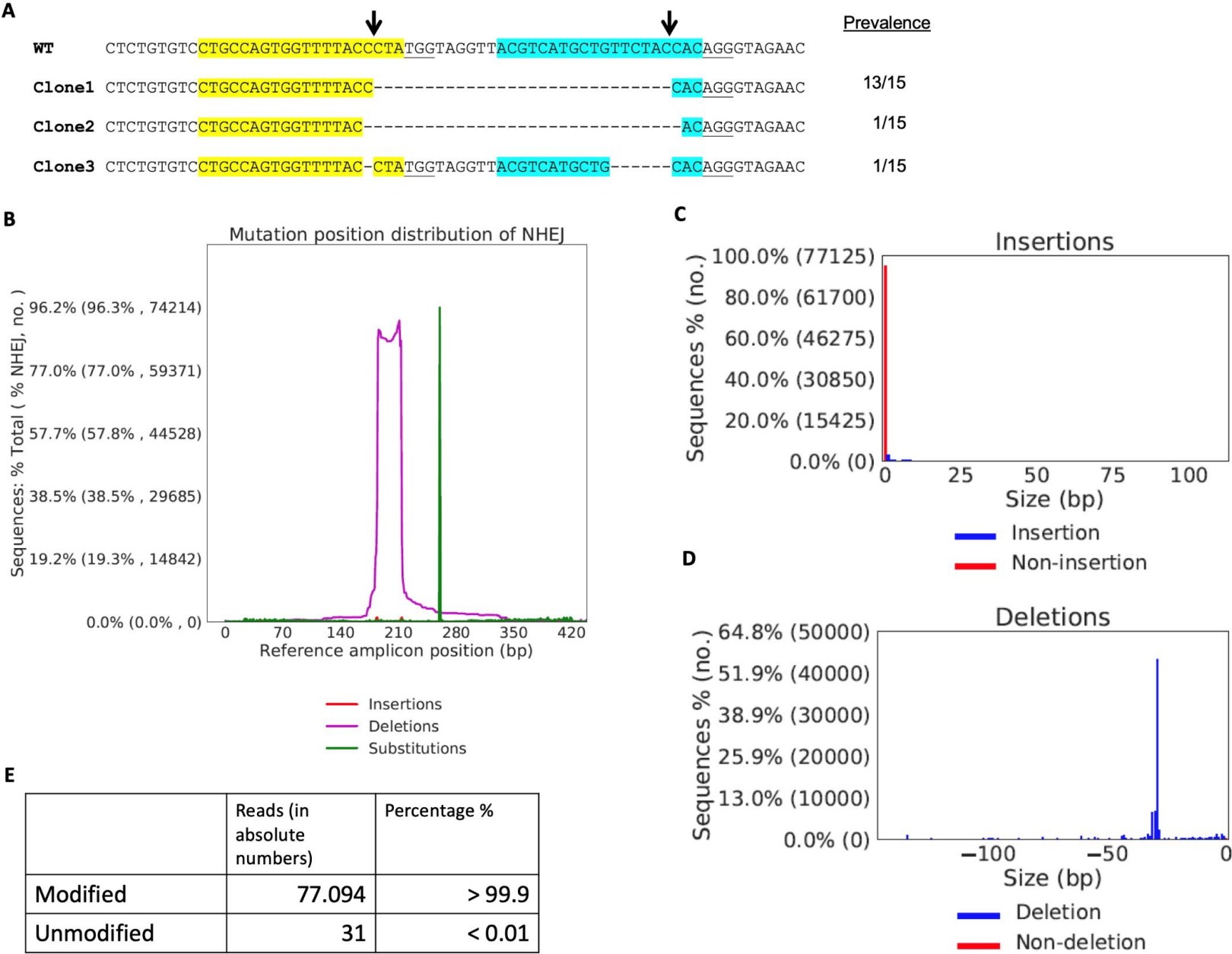

Supplementary Figure 4

Supplement: Multimedia component 4 [file mmc4.pdf]

**A**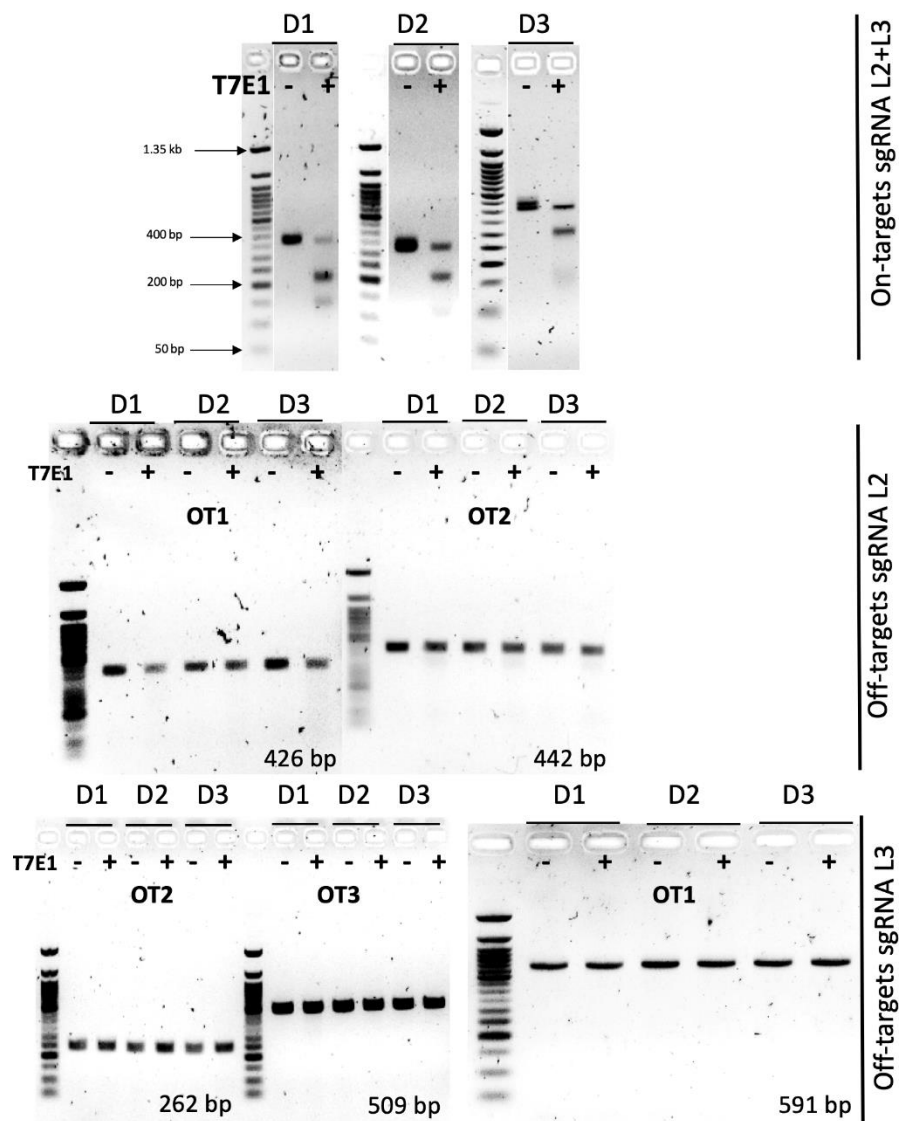**B**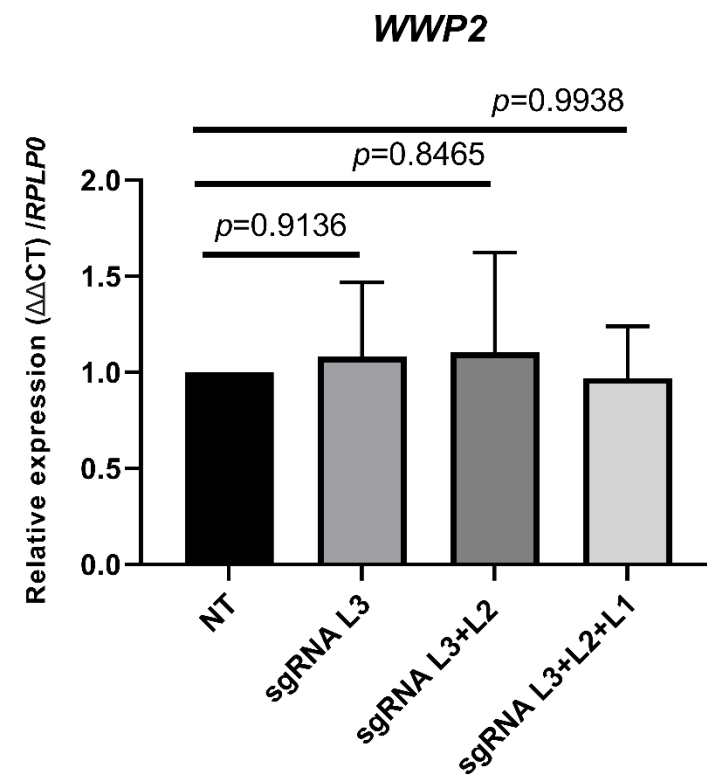

Supplementary Figure 5

Supplement: Multimedia component 5 [file mmc5.pdf]

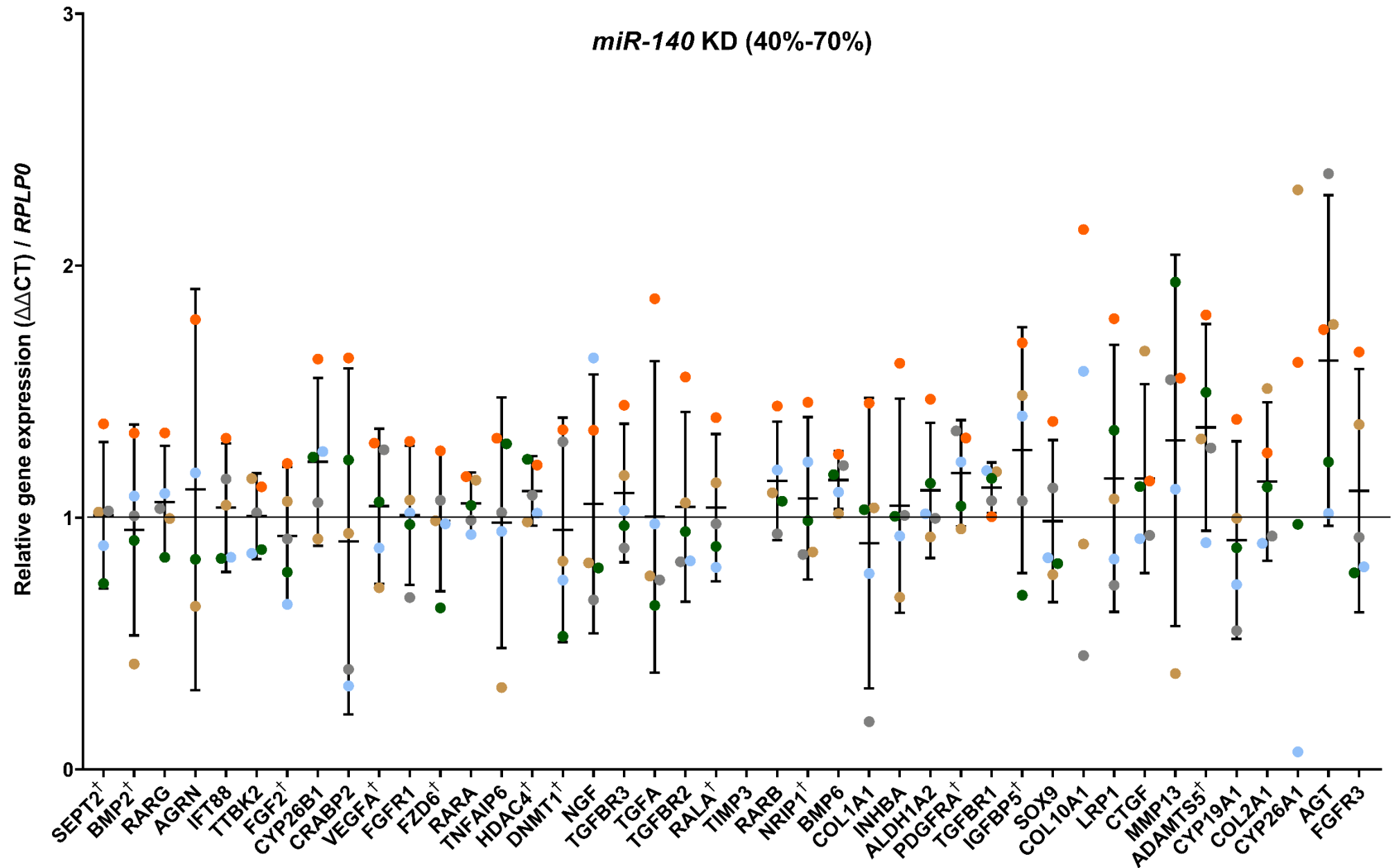

Supplementary Figure 6

Supplement: Multimedia component 6 [file mmc6.pdf]
