# Supplementary material for: Highly efficient CRISPR-Cas9-mediated editing identifies novel mechanosensitive microRNA-140 targets in primary human articular chondrocytes
Source: Osteoarthritis Cartilage. 2022 Apr;30(4):596–604. doi: 10.1016/j.joca.2022.01.005 (PMC8987936; doi:10.1016/j.joca.2022.01.005)
Supplement: Multimedia component 7 [file mmc7.docx]

| **Function** | **Genes** | **References** |
| --- | --- | --- |
| **Direct *miR-140* targets** | SEPT2, BMP2, FGF2, VEGFA, FZD6, HDAC4, DNMT1, RALA, NRIP1, PDGFRA, IGFBP5 | SEPT2: (1)  BMP2: (2)  FGF2: (3)  VEGFA: (4)  FZD6: (5)  HDAC4: (6)  DNMT1: (7)  RALA: (8)  NRIP1: (9)  PDGFRA: (10)  IGFBP5: (11) |
| **Cilia biology** | SEPT2, IFT88, TTBK2 | (1, 12)(Wann, unpublished) |
| **Retinoic acid** | RARG, CYP26B1, CRABP2, RARA, RARB, ALDH1A2, CYP19A1, CYP26A1, AGT | (13, 14) |
| **Anabolic genes** | BMP2, AGRN, FGF2, BMP6, COL1A1, SOX9, COL10A1, COL2A1 TNFAIP6, AGRN, TIMP3, COL1A1, INHBA, SOX9, COL10A1, LRP1, MMP13, ADAMTS5, COL2A1 | (2, 3, 15-20) |
| **Pain** | NGF | (21) |
| **Injury response** | FGF2, FGFR1, TGFA, TGFBR2, TGFBR3, TGFBR1, CTGF, FGFR3 | (22-24) |

**Supplementary Table 1**


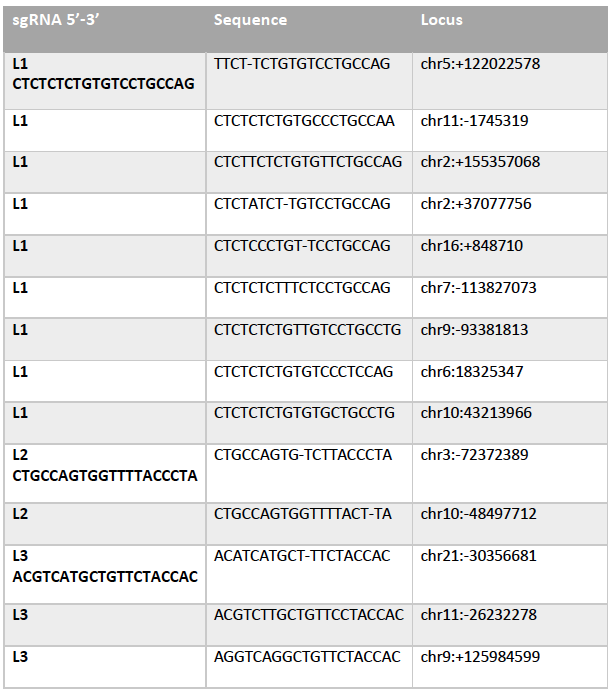


**Supplementary Table 2**

1. Yu J, Zhang W, Tang H, Qian H, Yang J, Zhu Z, et al. Septin 2 accelerates the progression of biliary tract cancer and is negatively regulated by mir-140-5p. *Gene.* 2016;589(1):20-6.

2. Hwang S, Park SK, Lee HY, Kim SW, Lee JS, Choi EK, et al. miR-140-5p suppresses BMP2-mediated osteogenesis in undifferentiated human mesenchymal stem cells. *FEBS Lett.* 2014;588(17):2957-63.

3. Ornitz DM, and Itoh N. The Fibroblast Growth Factor signaling pathway. *Wiley Interdiscip Rev Dev Biol.* 2015;4(3):215-66.

4. Lu Y, Qin T, Li J, Wang L, Zhang Q, Jiang Z, et al. MicroRNA-140-5p inhibits invasion and angiogenesis through targeting VEGF-A in breast cancer. *Cancer Gene Ther.* 2017;24(9):386-92.

5. Barter MJ, Tselepi M, Gomez R, Woods S, Hui W, Smith GR, et al. Genome-Wide MicroRNA and Gene Analysis of Mesenchymal Stem Cell Chondrogenesis Identifies an Essential Role and Multiple Targets for miR-140-5p. *Stem Cells.* 2015;33(11):3266-80.

6. Vega RB, Matsuda K, Oh J, Barbosa AC, Yang X, Meadows E, et al. Histone deacetylase 4 controls chondrocyte hypertrophy during skeletogenesis. *Cell.* 2004;119(4):555-66.

7. Takata A, Otsuka M, Yoshikawa T, Kishikawa T, Ohno M, and Koike K. MicroRNAs and liver function. *Minerva Gastroenterol Dietol.* 2013;59(2):187-203.

8. Karlsen TA, Jakobsen RB, Mikkelsen TS, and Brinchmann JE. microRNA-140 targets RALA and regulates chondrogenic differentiation of human mesenchymal stem cells by translational enhancement of SOX9 and ACAN. *Stem Cells Dev.* 2014;23(3):290-304.

9. Asano M, Umezu T, Katagiri S, Kobayashi C, Tauchi T, Gotoh M, et al. Up-regulated exosomal miRNA-140-3p in CML patients with musculoskeletal pain associated with discontinuation of tyrosine kinase inhibitors. *Int J Hematol.* 2017;105(4):419-22.

10. Lan H, Chen W, He G, and Yang S. miR-140-5p inhibits ovarian cancer growth partially by repression of PDGFRA. *Biomed Pharmacother.* 2015;75:117-22.

11. Tardif G, Hum D, Pelletier JP, Duval N, and Martel-Pelletier J. Regulation of the IGFBP-5 and MMP-13 genes by the microRNAs miR-140 and miR-27a in human osteoarthritic chondrocytes. *BMC Musculoskelet Disord.* 2009;10:148.

12. Coveney CR, Collins I, Mc Fie M, Chanalaris A, Yamamoto K, and Wann AKT. Cilia protein IFT88 regulates extracellular protease activity by optimizing LRP-1-mediated endocytosis. *FASEB journal : official publication of the Federation of American Societies for Experimental Biology.* 2018:fj201800334.

13. Zhu L, Chanalaris A, Groves K, Furniss D, Watt F, Gardiner M, et al. Polymorphic variants in ALDH1A2 determine the expression level of ALDH1A2 and CYP19A1 in the cartilage of patients undergoing trapeziectomy for severe thumb osteoarthritis. . *Osteoarthritis and Cartilage.* 2018;26.

14. Sparks MA, Crowley SD, Gurley SB, Mirotsou M, and Coffman TM. Classical Renin-Angiotensin system in kidney physiology. *Compr Physiol.* 2014;4(3):1201-28.

15. Eldridge SE, Barawi A, Wang H, Roelofs AJ, Kaneva M, Guan Z, et al. Agrin induces long-term osteochondral regeneration by supporting repair morphogenesis. *Sci Transl Med.* 2020;12(559).

16. Zhu L, Donhou S, Burleigh A, Miotla Zarebska J, Curtinha M, Parisi I, et al. TSG-6 Is Weakly Chondroprotective in Murine OA but Does not Account for FGF2-Mediated Joint Protection. *ACR Open Rheumatol.* 2020;2(10):605-15.

17. Little CB, Barai A, Burkhardt D, Smith SM, Fosang AJ, Werb Z, et al. Matrix metalloproteinase 13-deficient mice are resistant to osteoarthritic cartilage erosion but not chondrocyte hypertrophy or osteophyte development. *Arthritis and rheumatism.* 2009;60(12):3723-33.

18. Alexander S, Watt F, Sawaji Y, Hermansson M, and Saklatvala J. Activin A is an anticatabolic autocrine cytokine in articular cartilage whose production is controlled by fibroblast growth factor 2 and NF-kappaB. *Arthritis and rheumatism.* 2007;56(11):3715-25.

19. Oh CD, Lu Y, Liang S, Mori-Akiyama Y, Chen D, de Crombrugghe B, et al. SOX9 regulates multiple genes in chondrocytes, including genes encoding ECM proteins, ECM modification enzymes, receptors, and transporters. *PLoS One.* 2014;9(9):e107577.

20. Glasson SS, Askew R, Sheppard B, Carito B, Blanchet T, Ma H-L, et al. Deletion of active ADAMTS5 prevents cartilage degradation in a murine model of osteoarthritis. *Nature.* 2005;434(7033):644-8.

21. von Loga IS, El-Turabi A, Jostins L, Miotla-Zarebska J, Mackay-Alderson J, Zeltins A, et al. Active immunisation targeting nerve growth factor attenuates chronic pain behaviour in murine osteoarthritis. *Annals of the rheumatic diseases.* 2019;2019;78:672-5.

22. Tang X, Muhammad H, McLean C, Miotla-Zarebska J, Fleming J, Didangelos A, et al. Connective tissue growth factor contributes to joint homeostasis and osteoarthritis severity by controlling the matrix sequestration and activation of latent TGFβ. *Annals of the rheumatic diseases.* 2018.

23. Vincent T, Hermansson M, Bolton M, Wait R, and Saklatvala J. Basic FGF mediates an immediate response of articular cartilage to mechanical injury. *Proc Natl Acad Sci U S A.* 2002;99(12):8259-64.

24. Valverde-Franco G, Binette JS, Li W, Wang H, Chai S, Laflamme F, et al. Defects in articular cartilage metabolism and early arthritis in fibroblast growth factor receptor 3 deficient mice. *Human molecular genetics.* 2006;15(11):1783-92.
