# Supplementary material for: Highly efficient CRISPR-Cas9-mediated editing identifies novel mechanosensitive microRNA-140 targets in primary human articular chondrocytes
Source: Osteoarthritis Cartilage. 2022 Apr;30(4):596–604. doi: 10.1016/j.joca.2022.01.005 (PMC8987936; doi:10.1016/j.joca.2022.01.005)
Supplement: Multimedia component 8 [file mmc8.docx]

Supplementary methods

**Human chondrocyte isolation:**

Cartilage was cut into 4-8mm^3^ pieces, from which human articular chondrocytes were isolated by enzymatic digestion in Dulbecco’s modified eagle’s medium (DMEM) containing collagenase A (2 mg/ml) (Merck, Germany), 1 % penicillin, streptomycin, 20 ng/ml Fungizone and incubated between 18 h - 22 h at 37 °C with constant shaking. Digested material was passed through a 70 μm nylon mesh cell strainer and cells were centrifuged twice at 300g for 5 minutes in fresh medium, counted and cell viability assessed. They were seeded at 25,000 cells/cm2 and cultured at 37 °C, 20 % CO2 and 5 % CO2 in Dulbecco's modified Eagle's medium (DMEM) containing 4.5 g/l of glucose and L-Glutamine (Lonza, Verviers, Belgium), supplemented with 10 % fetal bovine serum (FBS) (Gibco, NY, USA).

**Murine Hip avulsion:** 5–6 week old C57Bl/6J mice were culled by CO_2_. The acetabulofemoral joint was exposed in a laminar flow hood and the hip joint was dislocated by applying pressure from behind the femur. The femoral cap was avulsed (shearing through the epiphyseal growth plate) using forceps. Hip cartilage was snap frozen (0 h) in liquid nitrogen or cultured in serum free medium for 4 h, and then frozen. Two hip joints from each mouse were pooled together and stored at -80°C. 8 mice were used in total. Hip cartilage was ground to a powder using Cryo-Cup Grinder (Biospec, Bartlesville, USA), and RNA was extracted using the RNeasy Mini kit (Qiagen, Hilden, Germany) according to the manufacturer’s instructions.

**Costal chondrocyte isolation and RNA-Seq from *miR-140*^-/-^ mice:** Primary mouse costal chondrocytes were isolation from 7-day-old *miR-140*^-/-^ and wild-type (WT) tissues as previously described ^32^. RNA was isolated using the miRVana miRNA Isolation Kit (Fisher Scientific) and sequenced after Illumina TruSeq Stranded mRNA sample preparation on an Illumina NextSeq500. Each sample provided >12 million single-end 75-bp sequencing reads. Sequenced reads were mapped to the mm10 transcriptome and analysed as previously described ^32^.

**pX330 Plasmid Transfection:**

To determine the optimal sgRNA and Cas9 integration agent, cells were either transfected with the pX330 plasmid, using Lipofectamine RNAiMAX (ThermoFisher Scientific), or in a ribonuclear protein (RNP) complex using Lipofectamine CRISPRMAX (ThermoFisher Scientific). For each, OA hACs were seeded into 12 well plates and transfected when they were 40%-60% confluent. Cells were visualized at 24 and 48h by light microscopy. Cell toxicity was measured using a Neubauer counting chamber of the trypan blue positive cells present in the supernatant after 48h. High cell death in the plasmid-transfected group led to all further experiments being performed using the RNP method.

**Genomic DNA (gDNA) and RNA extraction:** DNA and RNA extractions were performed 48h after transfection using AllPrep DNA/RNA mini kit from Qiagen (Hilden, Germany) according to the manufacturer’s instructions. RNA was on-column treated with DNase for 15 minutes. Genomic DNA and RNA samples were each eluted in 20 μl nuclease-free water.

**Sanger sequencing:** The region of interest was PCR amplified with the help of the Q5® Hot Start High-Fidelity Polymerase (New England Biolabs, Ipswich, MA, USA) which generates blunt ends. The subsequent PCR product was then transformed (with the help of the Zero Blunt TOPO PCR cloning kit, Invitrogen, Ipswich, MA, USA) into competent E. coli as per the manufacturer’s instructions and plated on lysogeny broth (LB) plates with kanamycin selection marker. 16 h post plating, 15 clones were picked and inoculated in LB + kanamycin media overnight. Cells were mini-prepped with the help of the QIAprep Spin Miniprep kit (Qiagen, Hilden, Germany) as per the manufacturer’s instructions, and sequenced by Eurofins Genomics, Germany.

**MiSeq Sequencing:** The approach requires two PCR reactions: the first using modified, locus specific primers (Fw 5’ ACACTCTTTCCCTACACGACGCTCTTCCGATCT- Target primer sequence (top strand) Rv: 5’ GACTGGAGTTCAGACGTGTGCTCTTCCGATCT- Target primer sequence (bottom strand)) using the Q5® Hot Start High-Fidelity Polymerase (New England Biolabs, Ipswich, MA, USA)). A second PCR amplification with Illumina sequencing adapters was performed as per the manufacturer’s instructions (NEBNext Multiplex Oligos for Illumina Index Primers, New England Biolabs, Ipswich, MA, USA). DNA was cleaned with AMPure XP beads (Beckman Coulter, Brea, CA, USA) and sequencing was performed using the MiSeq Reagent Kit V2 (San Diego, CA, USA) on an Illumina MiSeq Next Generation Sequencer (San Diego, CA, USA). Analysis was carried out by CRISPResso ^32^.

**microRNA quantification:** Stem-loop structure primers with specific sequences were obtained from Taqman (<https://www.thermofisher.com/order>) and used for microRNA RT-PCR (for *miR-140-5p* ID001187, for *miR-140-3p* ID002234). Prior to reverse transcription, the mature *miR* of choice was extended through *miR*-specific primers on each end through 5’ ligation and 3’ poly-A tailing of an adaptor sequence. These universal sequences on both end of the extended *miR* are then recognised by universal RT primers. Complementary DNA (cDNA) was synthesized from the DNase-treated RNA using a high capacity reverse transcription kit (Applied Biosystems, Massachusetts, USA). 15 ng RNA were added to a total of a 15 μl 1x master solution containing 1.5 μl of 10x RT buffer, 0.15 μl 10 mM deoxyribonucleotides (dNTPs), 0.19 μl RNase inhibitor (20 U), 1 μl reverse transcriptase (50 U), 1.5 μl 5x *miR*-specific RT primer and 1.5 μl 5x *miR*-specific housekeeper primer (RNU24), and 9.16 μl RNase free water per reaction using a C1000 touch thermocycler (BioRad, Hertfordshire, UK).

**Gene expression by microfluidic TaqMan assays:** A total of 10 μl of reaction mixture containing 0.7 μl cDNA template, 0.5 μl of 20x TaqMan® Small RNA Assay (containing probe), 5 μl 2x TaqMan universal PCR master mix (Applied Biosystems, Foster City, CA, USA) and 3.8 μl of nuclease-free water was added to each well of a 384 well plate. The plate was sealed and centrifuged for 1 min at 1500g. Real Time-qPCR was carried out on a ViiATM 7 Real-Time PCR System (Applied Biosystems) under the following conditions; 1X stage 1: 95 °C for 10 mins; 40X stage 2: 95 °C for 15 s; 60 °C for 1 min. Relative gene expression was calculated using the ΔΔCt method with *RPLP0* as the housekeeping gene.
